# Effects of a novel salt-tolerance *Lactiplantibacillus plantarum* on the physicochemical properties and volatile profiles of broad-bean paste

**DOI:** 10.1016/j.fochx.2026.103598

**Published:** 2026-01-26

**Authors:** Xiaoqi Gong, Wenjun Zuo, Xiaodan Tao, Zhijia Liu, Chuanqi Chu, Yujie Zhong, Tao Wang, Junjie Yi

**Affiliations:** a Faculty of Food Science and Engineering, Kunming University of Science and Technology, Kunming 650500, China; b Chuxiong Yunquan Sauce Co., Ltd., Chuxiong 675000, China; c Key Laboratory of Plateau Characteristic Prepared Food in Yunnan Province, Kunming 650500, China; d International Green Food Processing Research and Development Center of Kunming City, Kunming 650500, China

**Keywords:** *Lactiplantibacillus plantarum*, Broad bean paste, Starter culture, Flavor compound, Halotolerance

## Abstract

Broad bean paste (BBP) is a traditional Chinese condiment whose spontaneous fermentation often leads to unstable quality and safety concerns. This study isolated a novel halotolerant *Lactiplantibacillus plantarum* YQ1 from BBP as a starter culture. The strain exhibited exceptional salt tolerance (18% NaCl), acid and bile resistance, and a favorable biosafety profile. In BBP fermentation, it significantly reshaped metabolites, increasing tartaric and lactic acids, and promoting threonine accumulation. Volatile analysis demonstrated substantial improvements in desirable aroma compounds, with phenethyl alcohol increased by 436.37% and benzyl alcohol increased by 553%. Meanwhile, undesirable benzaldehyde, 3-methylbutanoic acids and 2-methylbutanoic acid decreased by 87%, 81%, and 62%. Genomic analysis further identified key genes underpinning these traits, as well as phenylalanine metabolism and alcohol-derived aroma biosynthesis. These findings highlight the potential of *L. plantarum* YQ1 as a starter culture to enhance the flavor, minimize off-flavors, and stabilize fermentation for high-salt BBP production.

## Highlights

• *L. plantarum* YQ1 exhibited exceptional salt (18% NaCl), acid, and bile tolerance.
• *L. plantarum* YQ1 fermentation increased phenethyl alcohol by 436%.
• *L. plantarum* YQ1 promoted beneficial metabolites like lactic acid and threonine.
• Genomics elucidated the mechanisms underlying stress tolerance and flavor formation.

## Graphical abstract

**Figure:**
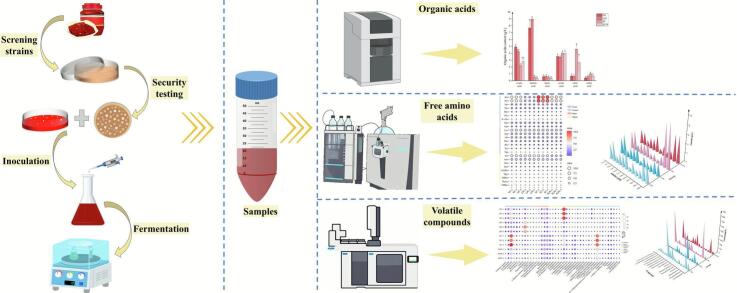

## 1 Introduction

Fermented bean products have gained significant attention due to their nutritional richness and distinctive sensory attributes. Broad bean paste (BBP, Doubanjiang), a traditional Chinese fermented condiment, is especially valued for its unique flavor characteristics and high protein content (Li et al., 2017). BBP is widely utilized in Chinese cuisine, significantly contributing to culinary traditions due to its complex taste and aromatic qualities. The production of BBP involves two crucial fermentation stages: koji-making and moromi fermentation. During the koji-making stage, steamed broad beans are mixed with wheat flour and inoculated with *Aspergillus oryzae*. This fungus proliferates rapidly and secretes various proteolytic enzymes, which hydrolyze proteins extensively and thereby enhance nutrient bioavailability. Subsequently, the mature koji undergoes moromi fermentation through the addition of high-concentration saline brine. This process fosters a unique microbial ecosystem that further transforms the substrates into diverse flavor and taste compounds (Niu et al., 2023).

Traditional BBP production largely depends on spontaneous, open-air fermentation, described as being “sun-dried by day and dew-moistened by night.” Although this practice fosters microbial diversity and enhances flavor complexity, it also results in considerable variability and inconsistent product quality. In addition, this process increases susceptibility to contamination by pathogenic or spoilage microorganisms and reduces overall product stability and safety (Tyagi et al., 2021). Consequently, it is necessary to identify and apply specific starter cultures, particularly probiotic strains, to guide flavor formation and inhibit spoilage organisms. These strains may also confer additional health benefits, making them especially valuable in BBP production.

Beyond the contributions of spices added during post-fermentation seasoning, the distinctive flavor profile of BBP predominantly originates from microbial metabolism during fermentation. The resident microbiota degrades macromolecules in broad beans (*e.g.*, proteins, starches, and lipids) into a diverse array of metabolites. These include volatile organic compounds (*e.g.*, alcohols, esters, and aldehydes) and taste-active substances like organic acids (OAs) and free amino acids (FAAs) (Jia et al., 2021; Yang et al., 2021; Zhang et al., 2020). These microbial metabolites collectively contribute to the characteristic savory aroma and the taste of BBP. Previous study has highlighted lactic acid bacteria (LAB) and yeast as the core functional microbiota driving BBP fermentation, underscoring their critical roles in shaping the final product quality (Zhang et al., 2020). Notably, *Lactiplantibacillus plantarum* has been recognized as a key LAB species closely linked to flavor formation in BBP (Zhao et al., 2024). Furthermore, LAB enhance both product safety and sensory attributes through the production of OAs, primarily lactic acid and acetic acid (Li, Guo, et al., 2023).

Given the crucial role of LAB in BBP fermentation, our preliminary study focused on isolating robust microbial strains with high salt tolerance to adapt to the saline characteristic of BBP production. Among the isolates, a novel salt-tolerant strain, *L. plantarum* YQ1, exhibited promising probiotic properties, including superior growth under high-salinity conditions and notable potential for flavor compound generation. Notably, compared with other reported halotolerant lactic acid bacteria, *L. plantarum* YQ1 not only grows under 18% NaCl but also exhibits a distinct metabolic profile. Specifically, it increases phenethyl alcohol by 436.37% during BBP fermentation, a yield substantially higher than that of common strains (Xue et al., 2024). Moreover, its genome is enriched with Na^+^/H^+^ antiporters and phenylalanine metabolism genes, providing a molecular basis for maintaining metabolic activity and flavor synthesis under high-salt conditions. Nevertheless, the practical application of this strain in BBP fermentation and its specific impact on product quality remain largely unexplored. Therefore, this study aims to investigate the potential of the *L.* *plantarum* YQ1 as a starter culture for BBP fermentation. Specifically, we will systematically evaluate its impact on the physicochemical characteristics, safety parameters, and volatile flavor compounds of BBP. Furthermore, we seek to characterize the genetic determinants of its metabolic functions, with particular emphasis on flavor-related biosynthetic pathways through whole-genome sequencing. The findings from this comprehensive investigation will contribute to the development of optimized fermentation strategies to improve product quality and process standardization in BBP production.

## 2 Materials and methods

### 2.1 Screening of salt-tolerant strains

In this study, a salt-tolerant strain (*L. plantarum* YQ1) was isolated from BBP samples obtained from Chuxiong Yunquan Sauce Co., Ltd. (Chuxiong, Yunnan, China). This strain has been deposited in the China General Microbiological Culture Collection Center (CGMCC, https://cgmcc.net/) under the accession number CGMCC No. 35267. Briefly, BBP samples were homogenized in sterile water and enriched in MRS broth supplemented with 10% (*w*/*v*) NaCl (≥ 99.0% purity, Damao Chemical Reagent Co., Ltd., Tianjin, China) at 37 °C for 3 d. The cultures were serially diluted and plated onto MRS agar plates containing 10% (*w*/*v*) NaCl, followed by incubation at 37 °C. Individual colonies were subsequently isolated, purified, and preliminarily identified through 16S rRNA gene sequencing. To further evaluate salt tolerance, purified isolates were cultured in MRS broth supplemented with 12%, 14%, 16%, or 18% (w/v) NaCl for 72 h. Viable cell counts (CFU/mL) were determined at the beginning of incubation (0 h) and after 72 h. The survival rate was defined as the logarithmic viable count at time t relative to the initial count, expressed as a percentage.

### 2.2 Strain identification by 16S rRNA gene sequencing

The salt-tolerant isolate was identified by 16S rRNA gene sequencing following a previously described method (Zhang et al., 2023) with minor modifications. Briefly, genomic DNA was extracted from cells harvested at the logarithmic growth phase. The 16S rRNA gene was amplified by PCR using a T100™ Thermal Cycler (BIO-RAD, Hercules, CA, USA). Sequencing was carried out by Sangon Biotechnology Co., Ltd. (Shanghai, China), and sequence similarity was analyzed using the BLAST database available at the National Center for Biotechnology Information (NCBI, http://www.ncbi.nlm.nih.gov/).

### 2.3 Strain characterization

#### 2.3.1 Hemolytic activity

Hemolytic activity was assessed according to the previous study (Li, Deng, et al., 2023). *L. plantarum* YQ1 was streaked onto blood agar plates containing defibrinated sheep blood (Huankai, Guangzhou, Guangdong, China) and incubated at 37 °C for 48 h. Zones of clearing around colonies were evaluated to classify hemolysis as α-hemolysis (greenish zone), β-hemolysis (clear zone), or γ-hemolysis (no zone). In this study, *Staphylococcus aureus* CICC10306 (obtained from the China Center of Industrial Culture Collection, Beijing, China) was used as a positive control strain.

#### 2.3.2 Antibiotic susceptibility

Antibiotic susceptibility was assessed using the disk diffusion assay (Shi et al., 2020), with some minor modifications. Suspensions of *L.* *plantarum* YQ1 were spread evenly onto MRS agar plates. Commercial antibiotic disks (BKMAM, Changsha, Hunan, China), including penicillin (10 U/piece), ampicillin (10 μg/piece), tetracycline (30 μg/piece), erythromycin (15 μg/piece), vancomycin (30 μg/piece), streptomycin (10 μg/piece), doxycycline (30 μg/piece), cefazolin (30 μg/piece), polymyxin B (300 μg/piece), and gentamicin (10 μg/piece). were placed onto the inoculated agar surface. Plates were incubated at 37 °C for 48 h. The susceptibility was interpreted according to the Clinical and Laboratory Standards Institute (CLSI) breakpoints.

#### 2.3.3 Acid and bile salt tolerance

Acid and bile salt tolerance were evaluated under simulated gastrointestinal conditions. Overnight cultures of *L.* *plantarum* YQ1 were inoculated (2% *v*/v) into MRS broth adjusted to pH 2.0, 2.5, or 3.0 with HCl (4 mol/L), or into MRS broth supplemented with oxgall bile salts (sodium taurocholate, 1, 2, 3, or 5 g/L; ≥ 60% purity, Yeasen Biotechnology Co., Ltd., Shanghai, China). Cultures were incubated at 37 °C with shaking at 200 rpm. Viable cell counts (CFU/mL) were determined at 0, 2, and 4 h by plating serial dilutions onto MRS agar. Survival rates were calculated as described in Section 2.1.

### 2.4 Whole-genome sequencing and analysis

Whole-genome sequencing of *L.* *plantarum* YQ1 was performed according to a previous study (Zhou et al., 2024). Briefly, bacterial cell pellets were collected and transported under dry ice to Majorbio Bio-pharm Technology Co., Ltd. (Shanghai, China) for genomic DNA extraction and sequencing. The functional annotation of the *L. plantarum* YQ1 genome was carried out with the aid of multiple bioinformatics resources. Protein sequences were analyzed against the Non-redundant Protein Database (NR), while metabolic pathway mapping was conducted using the Kyoto Encyclopedia of Genes and Genomes (KEGG). The KEGG database was primarily utilized to investigate the metabolic pathways involved in flavor compound generation from broad bean substrates by *L.* *plantarum* YQ1. A phylogenetic tree was constructed based on 31 housekeeping genes using the neighbor-joining method in MEGA software (v6.0).

### 2.5 Fermentation of broad bean samples

Fermentation broth obtained from Chuxiong Yunquan Sauce Co., Ltd. was used as the substrate. Specifically, the sample was collected at the fourth month of moromi fermentation (post-fermentation broth). This stage was selected because it is characterized by active microbial metabolism and substantial accumulation of flavor precursors, making it a suitable substrate for evaluating starter cultures. Moreover, using this partially fermented broth as the base can shorten the experimental fermentation period and facilitate a more rapid assessment of microbial effects. The salt content of the fermentation substrate was 16.27%. In this study, the post-fermentation broth was divided into four groups: (1) natural fermentation group (NF): unsterilized broth without inoculation; (2) *L. plantarum* YQ1 inoculated group (LPI): unsterilized broth inoculated with *L. plantarum* YQ1; (3) sterilized control group (SC): sterilized broth without inoculation; (4) sterilized *L. plantarum* YQ1 inoculated group (SLPI): sterilized broth inoculated with *L. plantarum* YQ1.

To minimize potential interference from the culture medium on BBP flavor, activated bacterial cells were harvested by centrifugation (3000 ×*g*, 5 min), washed twice with sterile water, and resuspended in sterile water. The optical density of the suspension at 600 nm (OD_600_) was adjusted to 0.6 ± 0.05, corresponding to approximately 10^8^ CFU/mL. Subsequently, 2% (*v*/v) of this suspension was inoculated into the LPI and SLPI groups. The sterilization condition is 121 °C for 15 min.

All groups were incubated at 37 °C with shaking at 200 rpm for 7 days. This duration was sufficient to clearly observe the preliminary effects of the strain on key metabolites and flavor components, while effectively avoiding metabolic stagnation caused by over-acidification or nutrient depletion. However, it should be noted that compared to the traditional BBP natural fermentation process, which can extend for several months to a year, this short-term experimental period is relatively limited and may not fully reveal the comprehensive role of the strain during prolonged fermentation. Therefore, in future study, it is necessary to design longer fermentation cycles to systematically investigate the sustained influence and regulatory mechanisms of *L. plantarum* YQ1 on quality development throughout the complete BBP fermentation process.

After fermentation, all fermented samples were stored at −80 °C for subsequent analyses. The sterilized groups (SC and SLPI) were specifically designed to evaluate the metabolic contributions of *L.* *plantarum* YQ1 in the absence of background microbiota. However, it should be noted that sterilization alters the physicochemical properties of the substrate, and thus these groups do not fully represent the native industrial fermentation ecology.

### 2.6 OAs analysis

OAs in BBP juice (FBPJ) were analyzed according to the method described previously (Yi et al., 2018). Briefly, FBPJ (1 mL) was deproteinized by adding 250 μL of potassium ferrocyanide (K₄[Fe(CN)₆], 150 g/L) and 250 μL of zinc sulfate (ZnSO₄, 300 g/L). After vortexing and standing at room temperature (∼25 ± 2 °C) for 30 min, the mixture was centrifuged (10,000 ×*g*, 4 °C, 20 min). The supernatant was filtered through a 0.22 μm membrane filter (Vander Biotechnology Co., Ltd., Beijing, China) and stored at −20 °C until analysis. Analysis was performed using high-performance liquid chromatography (HPLC) with a Prevail Organic Acid column (250 mm × 4.6 mm, 5 μm, Alltech Grace, Deerfield, FL, USA) at 30 °C. The mobile phase was 25 mM potassium phosphate buffer (pH 2.5) at a flow rate of 0.8 mL/min. Detection was performed using ultraviolet spectroscopy at 210 nm. OA standards (Tanmo Quality Testing Technology Co., Ltd., Hangzhou, China) were serially diluted to concentrations of 0.01, 0.05, 0.1, 0.2, 0.4, 0.6, and 0.8 mg/mL to establish a calibration curve for the qualitative and quantitative analysis of OA in FBPJ.

### 2.7 FAAs analysis

The content of FAAs in FBPJ was determined using ultra-high performance liquid chromatography-tandem mass spectrometry (UHPLC-MS/MS). Briefly, 50 μL of each sample was mixed with 200 μL of ice-cold extraction solvent (methanol: acetonitrile, 1:1 *v*/v) containing isotope-labeled internal standards. The mixture was vortexed for 30 s, sonicated in an ice-water bath for 15 min, stored at −40 °C for 1 h, and centrifuged (12,000 ×*g*, 4 °C, 15 min). The supernatant was diluted 20-fold with solvent, vortexed, and transferred into LC vials. Chromatographic separation was performed on a Waters ACQUITY UPLC BEH Amide column (100 mm × 2.1 mm, 1.7 μm, Waters, USA) at 35 °C. The mobile phase consisted of 1% formic acid in water (A) and 1% formic acid in acetonitrile (B). The injection volume was 1 μL. Detection was performed using an Agilent 6460 triple quadrupole mass spectrometer equipped with an AJS-ESI ion source operating in multiple reaction monitoring (MRM) mode. The ion source parameters were as follows: capillary voltage, +4000 V (positive) and − 3500 V (negative); nozzle voltage, +500 V and − 500 V; gas (N₂) temperature, 300 °C with a flow rate of 5 L/min; sheath gas (N₂) temperature, 250 °C with a flow rate of 11 L/min; and nebulizer pressure, 45 psi.

### 2.8 Flavor detection

#### 2.8.1 Electronic nose analysis

Volatile compounds were analyzed using a portable electronic nose system (cNose, Baosheng Industrial Development Co., Ltd., Shanghai, China) according to previous study (Gao et al., 2017). Briefly, 5 mL of FBPJ was transferred into a dedicated glass vial and equilibrated at 55.0 ± 0.5 °C for 15 min in a thermostatic water bath. Headspace gas was then introduced into the sensor chamber using an inert carrier gas at a constant flow rate of 1.0 L/min. The detection cycle lasted 120 s with continuous recording of sensor responses.

#### 2.8.2 Volatile compounds profiling and identification

Volatile compounds were further analyzed using headspace solid-phase microextraction coupled with gas chromatography–mass spectrometry (HS-SPME-GC/MS) (Wang et al., 2024). Briefly, 5 mL of FBPJ was mixed with 2.5 g NaCl and 10 μL of 3-octanol solution (100 μg/mL, internal standard) in a 20 mL SPME vial. After equilibration at 40 °C for 15 min, volatiles were extracted using a 50/30 μm DVB/CAR/PDMS SPME fiber (Supelco) at 40 °C for 40 min with agitation at 500 rpm. The fiber was then thermally desorbed in the GC injector port at 250 °C for 5 min in splitless mode. Separation was performed on a DB-5MS capillary column (30 m × 0.32 mm × 0.25 μm, Agilent Technologies, USA) with helium carrier gas at a constant flow of 3 mL/min. The oven temperature program was: initial temperature of 40 °C held for 3 min, ramped to 120 °C at 4 °C/min and held for 3 min, then increased to 230 °C at 10 °C/min and held for 3 min, followed by cooling back to 40 °C. Volatile compounds were identified by matching mass spectra with the NIST14 library and confirmed by calculating retention indices (RIs) relative to a C5-C25 n-alkane series analyzed under identical conditions. Quantification was based on the internal standard method.

### 2.9 Statistical analysis

All experiments were performed in triplicate. Data are expressed as mean ± standard deviation (SD). Statistical significance (*p* < 0.05) was evaluated using one-way analysis of variance (ANOVA) followed by Holm-Sidak's post-hoc test using Origin software (v2024, OriginLab). The bubble chart was generated by https://www.bioinformatics.com.cn, an online platform for data analysis and visualization.

## 3 Results and discussion

### 3.1 Identification and analysis of salt, acid, and bile salt tolerance in *L.plantarum* YQ1

**Fig. 1:**
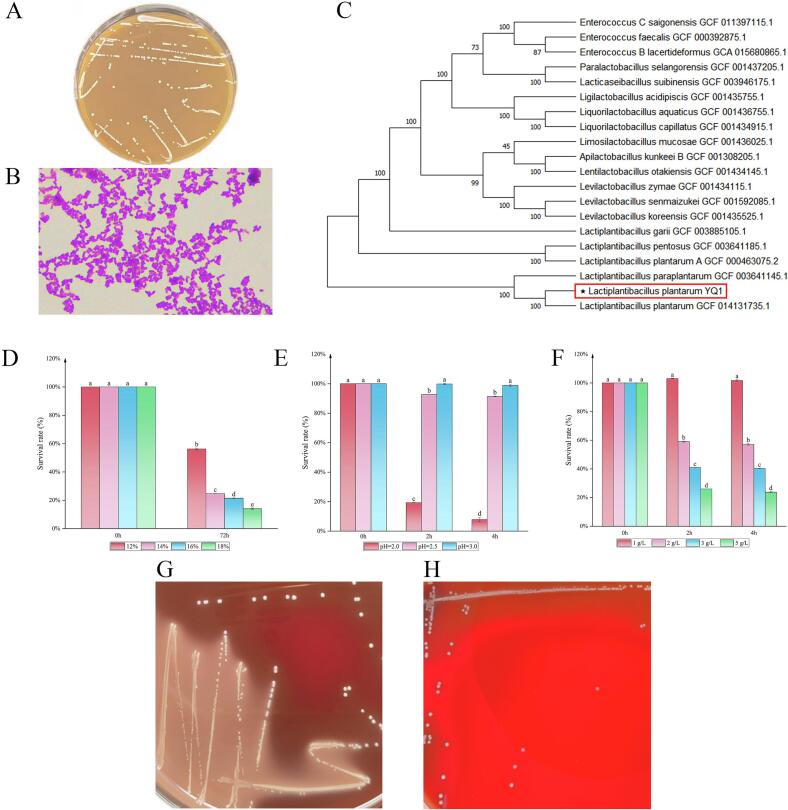
The biological characteristics and safety assessment of *L.plantarum* YQ1. (A) and (B), the representative colony morphology and Gram staining images of *L.plantarum* YQ1, respectively; (C), phylogenetic tree; (D), salt tolerance of *L.plantarum* YQ1; (E), acid resistance of *L.plantarum* YQ1; (F), bile salt tolerance of *L.plantarum* YQ1; (G), Hemolytic zone of *S. aureus* CICC10306 (positive control); (H), No hemolysis by *L.plantarum* YQ1.

A salt-tolerant bacterial strain was successfully isolated from BBP using MRS medium supplemented with 10% (*w*/*v*) NaCl. Colonies exhibited milky-white, circular, and moderately sized colonies, with smooth surfaces and regular edges (Fig. 1A). Gram staining and microscopic observation confirmed the isolate as a Gram-positive rod (Fig. 1B). 16S rRNA gene sequencing and subsequent phylogenetic analysis revealed 99.9% sequence similarity to *L.* *plantarum* (Fig. 1C).

The salt tolerance of *L. plantarum* YQ1 is shown in Fig. 1D. Remarkably, the strain sustained survival at 18% (w/v) NaCl, whereas reference strains such as *L.* *plantarum* D31 and T9 exhibited complete growth inhibition at NaCl concentrations exceeding 8.0% (Yao et al., 2020). This exceptional halotolerance can be attributed to the specific genomic adaptations in *L.* *plantarum* YQ1. Specifically, its genome encodes ten Na^+^/H^+^ antiporter genes, including five *napA*, four *nhaK*, and one *nhaC* homolog (Table 1). These transporters critically regulate cellular osmolarity by expelling sodium ions through proton exchange (Guo et al., 2017). In addition, three *kup* genes together with *trkA* and *trkH* (Table 1) in *L. plantarum* YQ1 can also mediate potassium uptake to maintain intracellular ion homeostasis (Chen et al., 2012). Complementary to these systems, cell envelope modification genes (*e.g.*, *plsY* and *mprF* for membrane remodeling, and *dapE*, *ltaS*, and *tagF* for cell wall biosynthesis, Table 1) can enhance structural integrity under osmotic stress (Bucka-Kolendo & Sokolowska, 2017). Collectively, these genomic features provide a molecular basis for the remarkable salt tolerance of *L.* *plantarum* YQ1 and underscore its potential as a robust culture for high-salt fermentation systems.

**Table 1:** The genetic information of *L.plantarum* YQ1.

Classify	Gene name	Function
Salt tolerance	*napA*	Na^+^/H^+^ antiporters
	*nhaK*	Na^+^/H^+^ antiporters
	*nhaC*	Na^+^/H^+^ antiporter NhaC
	*kup*	K^+^-transport system
	*trkA*	TrkA family potassium uptake protein
	*trkH*	Potassium transporter TrkG
	*plsY*	Glycerol-3-phosphate 1-O-acyltransferase PlsY
	*mprF*	Bifunctional lysylphosphatidylglycerol flippase/synthetase MprF
	*dapE*	ArgE/DapE family deacylase
	*ltaS*	LTA synthase family protein
	*tagF*	Teichoic acid biosynthesis protein
Acid tolerance	*atpA*	ATP synthase subunit beta, alpha, delta
	*atpD*	ATP synthase subunit beta, alpha, delta
	*atpH*	ATP synthase subunit beta, alpha, delta
	*argG*	Argininosuccinate synthase
	*ATP2C*	Calcium-transporting ATPase
Bile salt tolerance	*cbh*	Choloylglycine hydrolase
	*ppaC*	Manganese-dependent inorganic pyrophosphatase
	*yxeI*	Linear amide C—N hydrolase
	*pip*	Proline iminopeptidase

The acid tolerance of *L.* *plantarum* YQ1 under simulated gastrointestinal conditions is shown in Fig. 1E. Viability was markedly reduced at pH 2.0 (19.31% survival at 2 h and 8.08% at 4 h). However, survival remained high at pH 2.5 (> 92% at both 2 h and 4 h) and pH 3.0 (> 98%). Genome annotation also confirmed that this strain carries acid-resistant genes, such as *atpA*, *atpD*, *atpH*, *argG, ATP2C* (Table 1) (Zhao et al., 2019). These results demonstrate that *L.* *plantarum* YQ1 exhibits excellent acid tolerance, exceeding commonly accepted probiotic benchmarks, which require survival for at least 90 min at low pH and maintenance of viability at pH 3.0 for 2 h (Nguyen et al., 2023).

Bile salt tolerance was assessed at bile salt concentrations ranging from 1 to 5 g/L (Fig. 1F). Bacterial growth was unaffected at 1 g/L, but survival declined with increasing bile concentrations and exposure times. Notably, after 4 h at 3 g/L, a concentration approximating the human small intestine's average bile level (Parlindungan et al., 2021), *L. plantarum* YQ1 maintained a survival rate of 40.47%. Genome sequencing showed that this strain possesses at least four categories of bile salt-resistant genes, such as *cbh, ppaC, yxeI,* and *pip* (Table 1). This finding demonstrates that *L.* *plantarum* YQ1 possesses substantial bile tolerance, a key prerequisite for probiotic viability in the gastrointestinal tract (Sun et al., 2022; Zhang et al., 2022).

### 3.2 Bio-safety evaluation of *L.plantarum* YQ1

#### 3.2.1 Hemolytic activity

Hemolytic activity was assessed on sheep blood agar plates. The positive control, *S. aureus*, exhibited a clear zone indicative of β-hemolysis (Fig. 1G). In contrast, *L. plantarum* YQ1 colonies showed no visible zone of clearing or color change in the surrounding medium (Fig. 1H), indicating the absence of hemolytic activity and confirming a γ-hemolysis phenotype.

#### 3.2.2 Antibiotic resistance

The antibiotic susceptibility profile of *L.* *plantarum* is an essential parameter for assessing its safety as a potential starter culture (Koutsoumanis et al., 2021). As shown in Table 2, *L. plantarum* YQ1 was susceptible to penicillin, ampicillin, tetracycline, erythromycin, doxycycline, cefazolin, and gentamicin. However, resistance was observed against several antibiotics, including vancomycin, streptomycin, and polymyxin B. Genomic analysis further identified the corresponding resistance genes, including *vanT* and *vanH* for vancomycin, *rpsL* for streptomycin, *rosB* and *pmrF* for polymyxin B (Table 3). These findings are consistent with the intrinsic resistance traits commonly reported for *L.* *plantarum* (Jeong et al., 2021; Santamarina-García et al., 2024). Interestingly, unlike previous reports of natural cephalosporin resistance in *lactobacilli* (Anisimova & Yarullina, 2019), *L. plantarum* YQ1 was susceptible to cefazolin. Genomic analysis revealed only a partially covered β-lactamase gene (*LUT-6*, only 44.9% coverage), which is insufficient for functional expression, and no other cephalosporin-inactivating genes (*e.g.*, *TEM* and *SHV*) were detected. Overall, these findings suggest a low risk associated with the antibiotic resistance profile of *L.* *plantarum* YQ1 for potential applications.

**Table 2:** Sensitivity of *L.plantarum* YQ1 to antibiotics.

Antibiotic	Breakpoints (mm)			Inhibition zone diameter (mm)	Antibiotic resistancei)
	resistant	intermediate sensitive	sensitive		
Penicillin	≤ 14	–	≥ 15	24.1 ± 0.6	S
Ampicillin	≤ 13	14–16	≥ 17	33.9 ± 1.0	S
Tetracycline	≤ 14	15–18	≥ 19	22.6 ± 1.5	S
Erythromycin	≤ 13	14–22	≥ 23	34.7 ± 0.5	S
Vancomycin	≤ 14	15–16	≥ 17	ND	R
Streptomycin	≤ 11	12–14	≥ 15	ND	R
Doxycycline	≤ 12	13–15	≥ 16	28.8 ± 2.0	S
Cefazolin	≤ 13	14–16	≥ 17	34.0 ± 1.8	S
Polymyxin B	≤ 13	14–16	≥ 17	ND	R
Gentamicin	≤ 12	13–14	≥ 15	17.2 ± 1.2	S

Note: ND represents no inhibition zone observed.

i Antibiotic resistance: “S” represents sensitive; “R” represents resistant.

**Table 3:** Details of some drug resistance genes of *L.plantarum* YQ1.

Antibiotic	Genes	Mechanism	Identity (%)	Coverage (%)	*E*-value
Vancomycin	*vanT*	Antibiotic target alteration	31	97.3	1.11 × 10^−34^
	*vanH*	Antibiotic target alteration	33	95.8	1.05 × 10^−47^
Streptomycin	*rpsL*	Antibiotic target alteration	64	98.5	3.79 × 10^−55^
Polymyxin B	*rosB*	Antibiotic efflux	27.2	73.9	1.38 × 10^−14^
	*PmrF*	Antibiotic target alteration	27.6	99	1.15 × 10^−35^
Cefazolin	*LUT-6*	Antibiotic inactivation	29.5	44.9	9.66 × 10^−11^

Note: Based on sequence identity and coverage, genes with identity >70% and coverage >90% are likely functional homologs; identity 30–70% suggests potential functional similarity; identity <30% indicates low functional correlation. Coverage <50% suggests the gene fragment is unlikely to encode a functional protein.

### 3.3 OA content in FBPJ

**Fig. 2:**
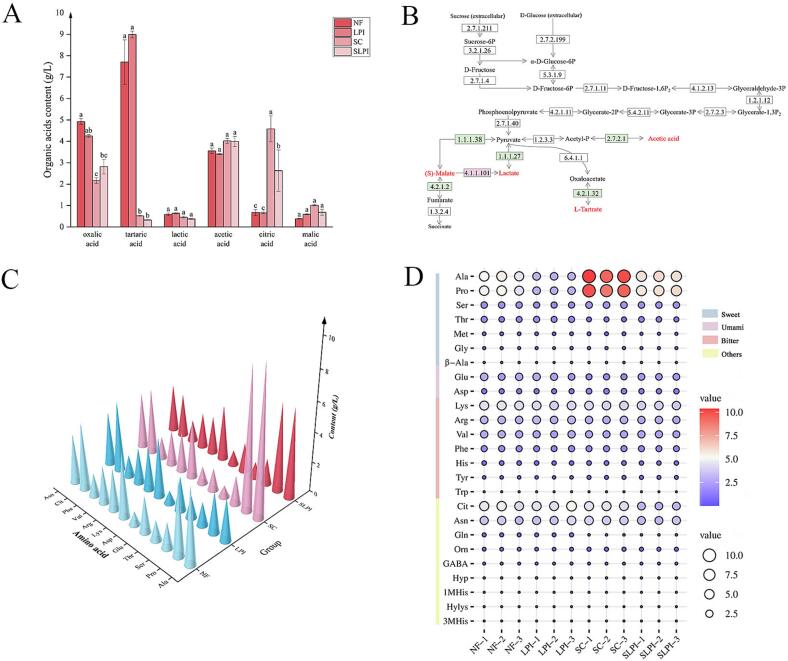
Analysis of organic acids and free amino acids in fermented broad bean paste juice and genomic prediction of organic acid metabolism in *L.plantarum* YQ1. (A), concentrations of organic acids; (B), proposed biosynthetic pathways for organic acids derived from genomic analysis; (C), bubble plot visualizing the composition and relative abundance of free amino acids; (D), column graph showing the concentrations of predominant amino acids. In the bubble plot, the bubble size and color encode the magnitude of the values, with larger sizes and redder colors indicating higher values, and smaller sizes and bluer colors indicating lower values, respectively.

LAB are well known for producing beneficial OAs such as lactic and acetic acids, which function not only as intermediates in biosynthetic and amino acid metabolism pathways but also as important modulators of product quality and safety. These acids contribute to the sensory balance of BBP and simultaneously inhibit the growth of spoilage organisms and pathogens during fermentation (Niu et al., 2024; Okoye et al., 2022; Yin et al., 2015). As shown in Fig. 2A, OA profiles in FBPJ varied significantly among the four treatment groups. The most pronounced divergence occurred between the non-sterilized (NF and LPI) and sterilized (SC and SLPI) systems. These differences primarily stemmed from substrate modification during high-temperature sterilization (121 °C, 15 min). While sterilized controls are useful for elucidating the intrinsic metabolic capacity of *L.* *plantarum* YQ1 under axenic conditions, their ecological relevance is limited. Sterilization disrupts the native microbial consortium and modifies the substrate matrix through protein denaturation and Maillard reactions, thereby decoupling strain behavior from the natural fermentation environment (Tang et al., 2021). Consequently, the SLPI group should be interpreted as reflecting monoculture physiology rather than community-driven fermentation dynamics. This distinction highlights the importance of evaluating strain performance under both simplified and complex ecological contexts when assessing its potential as a starter culture.

Among all treatments, tartaric, oxalic, and acetic acids consistently dominated the OA profile (Fig. 2A). Genomic analysis provided insights into the regulation of malate metabolism by *L.* *plantarum* YQ1 (Fig. 2B). Genes encoding malate dehydrogenase (EC 1.1.1.38) and fumarate hydratase (EC 4.2.1.2) of this strain enable reversible malate interconversion, whereas malate carboxylase (EC 4.1.1.101) catalyzes its irreversible degradation. This genetic framework explains the contrasting patterns observed: malate was elevated in LPI compared with NF but reduced in SLPI relative to SC. These results suggest that co-fermentation with *L.* *plantarum* YQ1 generally promotes malate accumulation, which may enhance acidity and freshness in the final product, a desirable trait in fermented seasonings. Similarly, the dynamics of lactate and tartrate could be attributed to strain-specific enzymes. The presence of L-lactate dehydrogenase (EC 1.1.1.27) and L(+)-tartrate dehydratase (EC 4.2.1.32) supports both synthesis and degradation, consistent with higher lactate and tartrate levels in LPI compared with NF, but lower levels in SLPI relative to SC. Such bidirectional regulation underscores the metabolic plasticity of *L.* *plantarum* YQ1, allowing it to adapt its acid metabolism depending on the ecological context. This ecological context-dependent metabolic plasticity and the resultant net flux are likely driven by microbial community interactions (Li et al., 2022). In the non-sterilized LPI system, *L. plantarum* YQ1 coexists with indigenous microbiota. Other community members may steer the catalytic reactions of malate dehydrogenase, lactate dehydrogenase, and tartrate dehydratase toward net synthesis, possibly by generating substantial amounts of organic acid precursors (*e.g.*, pyruvate) or secreting metabolic modulators that alter the intracellular cofactor balance (*e.g.*, NADH/NAD^+^) or the local micro-environment (*e.g.*, pH). Conversely, in the SLPI monoculture lacking community interactions, the isolated metabolic network may lead to feedback inhibition from accumulated products, resulting in the net consumption of these organic acids. Thus, the ultimate regulatory effect of *L.* *plantarum* YQ1 on the organic acid profile is an outcome of its inherent bidirectional enzyme systems coupled with the complex microbial interactions within a specific fermentation niche (Qian et al., 2021).

Interestingly, no direct oxalate- or citrate-specific metabolic genes were identified in the genome of *L.* *plantarum* YQ1. This observation suggests that the effects of inoculation on oxalic and citric acid levels may be indirect, potentially arising from altered precursor fluxes or microbial interactions within the fermentation consortium. Given that oxalate accumulation is often associated with undesirable sensory and nutritional consequences, the apparent ability of *L.* *plantarum* YQ1 to modulate these levels indirectly may represent an additional advantage in terms of product quality.

### 3.4 FAA composition in FBPJ

FAAs are important contributors to the overall flavor of fermented foods, functioning both as direct taste compounds and as precursors of key aroma molecules (Fernandez & Zuniga, 2006). In BBP fermentation, FAAs undergo biochemical transformations such as Maillard reaction and Strecker degradation, leading to the formation of volatile flavor components, including aldehydes, ketones, esters, and pyrazines (Chen, Chen, et al., 2020). Based on their taste attributes, FAAs are generally classified as sweet-tasting (*e.g.*, threonine, serine, glycine, alanine, proline, and methionine), umami-tasting (*e.g.*, glutamic acid and aspartic acid), and bitter-tasting (*e.g.*, histidine, lysine, valine, tryptophan, tyrosine, phenylalanine, and arginine) (Liang et al., 2025; Lin et al., 2018; Zhao et al., 2016).

During BBP fermentation, microbial metabolism markedly influenced FAA dynamics. As shown in Table S1 and Fig. 2C-D, sweet- and bitter-tasting FAAs accounted for the majority of the FAA pool. Inoculation with *L. plantarum* YQ1 resulted in a clear reduction in the total concentration of these FAAs, while umami-tasting FAAs remained relatively stable. This pattern suggests that *L.* *plantarum* YQ1 not only actively metabolizes amino acids but may also reshape their turnover through interactions with the resident microbiota, thereby shifting the FAA balance in ways that could ultimately affect flavor development. Among individual FAAs, threonine showed a remarkable increase in both *L. plantarum* YQ1-inoculated groups (LPI and SLPI) compared to their uninoculated controls (NF and SC) (Table S1). Genomic analysis indicated that *L. plantarum* YQ1 possesses genes encoding threonine synthase (EC 4.2.3.1, *thrC*) in the glycine-serine-threonine pathway (KEGG map00260), but lacks genes for key degradative enzymes such as L-threonine ammonia-lyase (EC 4.3.1.19, *SDS*). This genetic profile explains the accumulation of threonine, suggesting that *L. plantarum* YQ1 can synthesize it *de novo* while limiting its catabolism. Such a capability not only drives threonine retention in monoculture (SLPI) but also appears to prevent its depletion in more complex microbial systems (LPI). This dual effect underscores the ecological competitiveness of *L. plantarum* YQ1 and its potential contribution to the sensory quality of BBP.

Beyond their direct taste contributions, FAAs also function as key precursors for diverse aroma formation (Lin et al., 2019). For instance, phenylalanine plays a pivotal role in the biosynthesis of aromatic volatiles. Through transamination catalyzed by aromatic amino acid transaminases (EC 2.6.1.57), phenylalanine is converted into phenylpyruvate, which is subsequently decarboxylated by aromatic amino acid decarboxylases (EC 4.1.1.53) to form phenylacetaldehyde. Reduction of this intermediate yields phenethyl alcohol, an abundant contributor to the characteristic aroma profile of BBP. Thus, the dynamic shifts in FAA composition not only alter the basic taste profile but also shape the aromatic complexity of the final product.

### 3.5 Volatile compound profiling and analysis in FBPJ

#### 3.5.1 Electronic nose analysis

**Fig. 3:**
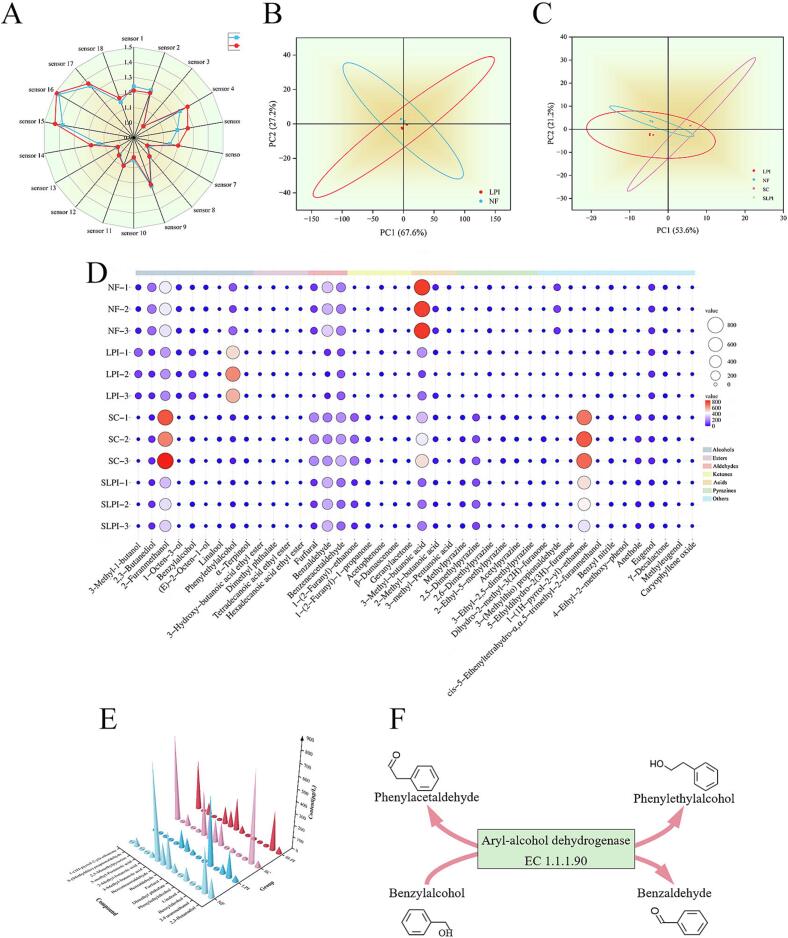
Volatile compound profiles and proposed biosynthetic pathway. (A), radar chart of electronic nose sensor responses; (B), PCA of electronic nose data; (C), PCA of flavor compound; (D), bubble plot of identified volatile compounds; (E), concentration histogram of key volatiles; (F), schematic representation of phenethyl alcohol and benzaldehyde biosynthetic pathways. In the bubble plot, the bubble size and color encode the magnitude of the values, with larger sizes and redder colors indicating higher values, and smaller sizes and bluer colors indicating lower values, respectively.

Electronic nose technology, which mimics human olfaction using sensor arrays to detect overall odor profiles, was employed to rapidly distinguish aroma differences between fermentation groups (Sun et al., 2025). The radar chart (Fig. 3A) revealed that group NF exhibited higher responses in sensor 1 (short-chain alkanes), sensor 2 (broad carbon compounds), and sensor 10 (hydrogen) compared with LPI. However, responses from all other sensors were lower in NF than in LPI. This pattern indicates that inoculation with *L.* *plantarum* YQ1 markedly altered the volatile composition. Principal component analysis (PCA) of the electronic nose data (Fig. 3B) showed that the first two principal components (PC1 and PC2) explained 94.8% of the total variance (PC1: 67.6%; PC2: 27.2%). The PCA plot clearly separated groups NF and LPI, indicating fundamental differences in their volatile composition. Collectively, the radar chart and PCA results suggest that inoculation with *L. plantarum* YQ1 resulted in a distinct and potentially more favorable overall flavor profile, as perceived by the electronic nose sensor array.

#### 3.5.2 GC–MS detection and analysis

The volatile compounds identified in FBPJ by GC–MS are summarized in Table S2. Compared to the uninoculated control (NF group), inoculation with *L. plantarum* YQ1 (LPI group) markedly altered the volatile profile of FBPJ (Fig. 3C-D). As shown in Fig. 3E, several compounds, predominantly associated with off-flavors, showed decreased levels following fermentation with *L.* *plantarum* YQ1. These included 2,3-butanediol (buttery), 2-furanmethanol (caramelized), benzaldehyde (bitter almond), phenylacetaldehyde (bitter almond), acetophenone (bitter almond), 3-methylbutanoic acid (pungent, sour), 2-methylbutanoic acid (cheese-like), 3-methylpentanoic acid (rancid, sour), and 3-(methylthio) propanal (potato-like). At the same time, several desirable compounds increased in the LPI group, such as 3-methyl-1-butanol (fruity), benzyl alcohol (floral), phenethyl alcohol (rosy), and dimethyl phthalate. Moreover, entirely new volatiles were detected after fermented with *L.* *plantarum* YQ1, including linalool (citrus), ethyl tetradecanoate (floral/fruity), and caryophyllene oxide (woody). Collectively, these shifts suggest that *L. plantarum* YQ1 not only degrades or transforms pre-existing volatiles but also contributes to the synthesis of new compounds, likely through complex interactions with the resident microbiota (Xue et al., 2025).

Among the key aroma-active molecules, four compounds with significant changes (phenylacetaldehyde, phenethyl alcohol, benzaldehyde, and benzyl alcohol) were particularly influential in shaping the BBP flavor profile. Phenylacetaldehyde is a characteristic contributor to the aroma of traditional fermented soybean pastes, whereas phenethyl alcohol, benzaldehyde, and benzyl alcohol exert significant effects on the overall aroma (Lin et al., 2019; Zhao et al., 2011). Notably, despite their contribution to the unique sensory attributes of fermented foods, benzaldehyde is associated with bitter-almond or pungent odors, which may compromise product quality when present above sensory thresholds (Chen, Zhou, et al., 2020). In this study, fermentation with *L. plantarum* YQ1 induced distinct changes in these compounds. In the LPI group, the concentration of phenethyl alcohol increased dramatically by 436.37% (579.82 μg/L) relative to the NF group (108.10 μg/L). Conversely, both phenylacetaldehyde and benzaldehyde decreased markedly, by 40.16% (LPI: 110.87 μg/L *vs.* NF: 185.28 μg/L) and 86.98% (LPI: 40.35 μg/L *vs.* NF: 309.98 μg/L), respectively. Furthermore, co-inoculation with *L.* *plantarum* YQ1 (LPI) markedly enhanced benzyl alcohol accumulation (553.67% increase) while concomitantly reducing benzaldehyde concentration. In contrast, under sterilized monoculture conditions (SLPI *vs.* SC), *L. plantarum* YQ1 exhibited the opposite trend, with a significant increase in benzaldehyde coupled to a reduction in benzyl alcohol (Fig. 3E). These findings indicate that *L.* *plantarum* YQ1 promotes the formation of favorable aroma-active alcohols and suppresses the accumulation of undesirable aldehydes in the context of complex microbial communities, whereas under monoculture conditions, it irreversibly drives the conversion of benzyl alcohol into benzaldehyde (Guo et al., 2024; Hu et al., 2025). This ecological context-dependent shift in metabolic outcomes may be attributed to several interconnected factors (Chen et al., 2024). Within a complex consortium, cross-feeding interactions—such as the supply of intermediate metabolites by other microorganisms (*e.g.*, yeasts) or the removal of inhibitory products—could redirect *L.* *plantarum* YQ1's metabolic flux toward phenethyl alcohol synthesis. In contrast, under pure culture conditions, reduced substrate competition and the absence of community-level metabolic regulation may favor benzaldehyde accumulation as a terminal metabolite. Furthermore, environmental parameters that differ between the two systems, including pH dynamics, redox potential, and the presence of quorum-sensing molecules (Yang et al., 2025), could modulate the activity or expression of key enzymes such as aryl-alcohol dehydrogenase, thereby influencing the final aroma profile.

From a mechanistic perspective, these transformations can be explained through phenylalanine metabolism (KEGG map00360) and toluene degradation pathway (KEGG map00623). Phenethyl alcohol is synthesized from phenylalanine *via* phenylpyruvate and phenylacetaldehyde, while benzyl alcohol and benzaldehyde are interconverted through the toluene degradation pathway. Central to these reactions is aryl-alcohol dehydrogenase (EC 1.1.1.90), encoded in the genome of *L.* *plantarum* YQ1, which catalyzes the reversible reduction of phenylacetaldehyde to phenethyl alcohol as well as the interconversion between benzyl alcohol and benzaldehyde (Fig. 3F). The expression of this gene in *L.* *plantarum* YQ1 provides direct mechanistic evidence linking its enzymatic activity to the observed shifts in volatile profiles. Collectively, these results underscore the context-dependent metabolic behavior of L. *plantarum* YQ1, which modulates key aroma compounds differently depending on whether it is embedded within a complex microbial consortium or functioning in isolation.

Overall, both electronic nose and GC–MS analyses demonstrated that inoculation with *L.* *plantarum* YQ1 profoundly reshaped the volatile profile of FBPJ. Inoculated samples were clearly separated from the control, characterized by reduced levels of compounds linked to off-flavors (*e.g.*, rancid and putrid notes) and increased concentrations of desirable aroma compounds (*e.g.*, floral and fruity notes). In addition, new floral, fruity, and woody volatiles such as linalool and caryophyllene oxide were detected only in the LPI group. Collectively, these results indicate that *L.* *plantarum* YQ1 enhances the overall aroma quality of FBPJ by suppressing undesirable volatiles while promoting the accumulation and generation of favorable compounds.

## 4 Conclusions

In this study, a novel halotolerant strain, *L. plantarum* YQ1, was successfully isolated from traditional BBP and comprehensively characterized. The strain exhibited exceptional salt, acid, and bile tolerance, as well as a favorable biosafety profile, underscoring its potential as a robust starter culture for high-salt fermentations. Application of *L.* *plantarum* YQ1 in BBP fermentation significantly reshaped the metabolite landscape: organic acid and amino acid analyses highlighted enhanced threonine accumulation and elevated lactic acid production, while volatile profiling (*E*-nose and GC–MS) demonstrated a clear shift toward desirable floral and fruity aroma compounds accompanied by the reduction of off-flavor aldehydes and acids. Genomic evidence further revealed the metabolic pathways underlying these transformations, particularly phenylalanine- and alcohol-derived flavor biosynthesis. Collectively, these results establish *L.* *plantarum* YQ1 as a promising functional starter culture capable of improving both the sensory quality and consistency of BBP. Beyond its role in traditional condiment production, this strain provides a valuable model for understanding LAB-driven metabolic modulation under high-salinity conditions. Future work should focus on pilot-scale validation, sensory evaluation by consumer panels, and exploration of synergistic effects with other core microbiota to facilitate industrial application and standardization of BBP fermentation.

## CRediT authorship contribution statement

**Xiaoqi Gong:** Writing – original draft, Methodology, Investigation. **Wenjun Zuo:** Methodology, Investigation. **Xiaodan Tao:** Methodology, Investigation. **Zhijia Liu:** Funding acquisition, Data curation. **Chuanqi Chu:** Validation, Formal analysis. **Yujie Zhong:** Writing – review & editing, Software. **Tao Wang:** Writing – review & editing, Project administration, Funding acquisition. **Junjie Yi:** Supervision, Funding acquisition, Conceptualization.

## Fundings

This work was financially supported by the Yunnan Academician Expert Workstation (No. 202305AF150039), Yunnan Fundamental Research Projects (No. 202201BE070001–005 and No. 202401CF070092).

## Declaration of competing interest

The authors declare that they have no known competing financial interests or personal relationships that could have appeared to influence the work reported in this paper.

## Data Availability

Data will be made available on request.

## Appendix A Supplementary data

Supplementary material Image 1

## References

[bb0005] Anisimova E.A., Yarullina D.R. (2019). Antibiotic resistance of *Lactobacillus* strains. Current Microbiology.

[bb0010] Bucka-Kolendo J., Sokolowska B. (2017). Lactic acid bacteria stress response to preservation processes in the beverage and juice industry. Acta Biochimica Polonica.

[bb0015] Chen C., Ai L., Zhou F., Ren J., Sun K., Zhang H., Chen W., Guo B. (2012). Complete nucleotide sequence of plasmid pST-III from *Lactobacillus plantarum* ST-III. Plasmid.

[bb0020] Chen C., Zhou W., Yu H., Yuan J., Tian H. (2020). Evaluation of the perceptual interactions among aldehydes in a cheddar cheese matrix according to odor threshold and aroma intensity. Molecules.

[bb0025] Chen D.L., Qu Z.P., Yang S.J., Li Y.J., Yu S.X., Li X., Qiao J.J. (2024). Quorum sensing regulating the productivity and stability of cross-feeding cocultivation. Chemical Engineering Journal.

[bb0030] Chen G., Chen Y., Hou Y., Huo Y., Gao A., Li S., Chen Y. (2020). Preparation, characterization and the in vitro bile salts binding capacity of celery seed protein hydrolysates via the fermentation using *B. Subtilis*.. LWT-Food Science and Technology.

[bb0035] Fernandez M., Zuniga M. (2006). Amino acid catabolic pathways of lactic acid bacteria. Critical Reviews in Microbiology.

[bb0040] Gao L., Liu T., An X., Zhang J., Ma X., Cui J. (2017). Analysis of volatile flavor compounds influencing Chinese-type soy sauces using GC-MS combined with HS-SPME and discrimination with electronic nose. Journal of Food Science and Technology-Mysore.

[bb0045] Guo Q., Peng J., He Y. (2024). A systematic comparative study on the physicochemical properties, volatile compounds, and biological activity of typical fermented soy foods. Foods.

[bb0050] Guo Y., Tian X., Huang R., Tao X., Shah N.P., Wei H., Wan C. (2017). A physiological comparative study of acid tolerance of *Lactobacillus plantarum* ZDY 2013 and *L-plantarum* ATCC 8014 at membrane and cytoplasm levels. Annals of Microbiology.

[bb0055] Hu K.D., Li J.L., Du Y.Q., Zhang M., Wang X.J., Wan Y.X., Liu S.L. (2025). Aroma-producing *Lactiplantibacillus plantarum* LYA31 and *Wickerhamomyces anomalus* YYB24: Insight into their contribution to paocai fermentation. LWT- Food Science and Technology.

[bb0060] Jeong C.-H., Sohn H., Hwang H., Lee H.-J., Kim T.-W., Kim D.-S., Hong S.-W. (2021). Comparison of the probiotic potential between *Lactiplantibacillus plantarum* isolated from kimchi and standard probiotic strains isolated from different sources. Foods.

[bb0065] Jia Y., Niu C.-T., Xu X., Zheng F.-Y., Liu C.-F., Wang J.-J., Lu Z.-M., Xu Z.-H., Li Q. (2021). Metabolic potential of microbial community and distribution mechanism of *Staphylococcus* species during broad bean paste fermentation. Food Research International.

[bb0070] Koutsoumanis K., Allende A., Alvarez-Ordonez A., Bolton D., Bover-Cid S., Chemaly M., Hazards E.P.B. (2021). Role played by the environment in the emergence and spread of antimicrobial resistance (AMR) through the food chain. EFSA Journal.

[bb0075] Li H., Deng W., Lu Z.-M., Li X., Fan Z., Zhang Q., Chen G., Li Q., Ma Y., Xu Z.-H. (2023). Salinity plays a dual role in broad bean paste-meju fermentation. LWT- Food Science and Technology.

[bb0080] Li S., Guo L., Gu J., Mu G., Tuo Y. (2023). Screening lactic acid bacteria and yeast strains for soybean paste fermentation in northeast of China. Food Science & Nutrition.

[bb0085] Li W.L., Tong S.G., Yang Z.Y., Xiao Y.Q., Lv X.C., Weng Q., Ni L. (2022). The dynamics of microbial community and flavor metabolites during the acetic acid fermentation of Hongqu aromatic vinegar. Current Research in Food Science.

[bb0090] Li Z., Rui J., Li X., Li J., Dong L., Huang Q., Huang C., Wang Z., Li L., Xuan P., Tang Y., Chen F. (2017). Bacterial community succession and metabolite changes during doubanjiang-meju fermentation, a Chinese traditional fermented broad bean paste. Food Chemistry.

[bb0095] Liang J., Wang Y., Wang T., Chu C., Yi J., Liu Z. (2025). Enhancing fermented vegetable flavor with *Lactobacillus plantarum* and *Rhodotorula mucilaginosa*. Food Research International.

[bb0100] Lin H., Yu X., Fang J., Lu Y., Liu P., Xing Y., Wang Q., Che Z., He Q. (2018). Flavor compounds in pixian broad-bean paste: Non-volatile organic acids and amino acids. Molecules.

[bb0105] Lin H.B., Liu Y., He Q., Liu P., Che Z.M., Wang X.M., Huang J.Q. (2019). Characterization of odor components of Pixian Douban (broad bean paste) by aroma extract dilute analysis and odor activity values. International Journal of Food Properties.

[bb0110] Nguyen N.H.K., Giang B.L., Truc T.T. (2023). Isolation and evaluation of the probiotic activity of lactic acid bacteria isolated from pickled *Brassica juncea* (L.) Czern. Et Coss. Foods.

[bb0115] Niu C., Xing X., Wang Y., Li X., Zheng F., Liu C., Wang J., Li Q. (2023). Characterization of color, metabolites and microbial community dynamics of doubanjiang during constant temperature fermentation. Food Research International.

[bb0120] Niu C., Xing X., Zuo W., Zuo Z., Liu F., Liu C., Li Q. (2024). Construction and application of a synthetic microbial community in reduced salinity fermentation of raw-materials based broad bean paste. Food Bioscience.

[bb0125] Okoye C.O., Dong K., Wang Y.L., Gao L., Li X., Wu Y.F., Jiang J.X. (2022). Comparative genomics reveals the organic acid biosynthesis metabolic pathways among five lactic acid bacterial species isolated from fermented vegetables. New Biotechnology.

[bb0130] Parlindungan E., Lugli G.A., Ventura M., van Sinderen D., Mahony J. (2021). Lactic acid bacteria diversity and characterization of probiotic candidates in fermented meats. Foods.

[bb0135] Qian W., Lu Z.M., Chai L.J., Zhang X.J., Li Q., Wang S.T., Xu Z.H. (2021). Cooperation within the microbial consortia of fermented grains and pit mud drives organic acid synthesis in strong-flavor baijiu production. Food Research International.

[bb0140] Santamarina-García G., Amores G., Llamazares D., Hernández I., Barron L.J.R., Virto M. (2024). Phenotypic and genotypic characterization of antimicrobial resistances reveals the effect of the production chain in reducing resistant lactic acid bacteria in an artisanal raw ewe milk PDO cheese. Food Research International.

[bb0145] Shi Y., Zhai M., Li J., Li B. (2020). Evaluation of safety and probiotic properties of a strain of *enterococcus faecium* isolated from chicken bile. Journal of Food Science and Technology-Mysore.

[bb0150] Sun Y.C., Yu G., Lu Q., Han H.X., Yang J.W., Xu Y.Q. (2025). An electronic nose device with rapid and universal odor detection capability. Sensors and Actuators B-Chemical.

[bb0155] Sun Y.W., Zhang S.Y., Li H., Zhu J., Liu Z.J., Hu X.S., Yi J.J. (2022). Assessments of probiotic potentials of *Lactiplantibacillus plantarum* strains isolated from chinese traditional fermented food: Phenotypic and genomic analysis. Frontiers in Microbiology.

[bb0160] Tang J.J., Zhang Z.X., Zheng S.L., Gao N., Li Z.J., Li K. (2021). Changes of main nutrient components and volatile flavor substances in processing of canned bamboo shoots. Fermentation-Basel.

[bb0165] Tyagi A., Shabbir U., Chelliah R., Daliri E.B.-M., Chen X., Oh D.-H. (2021). *Limosilactobacillus reuteri* fermented brown rice: A product with enhanced bioactive compounds and antioxidant potential. Antioxidants.

[bb0170] Wang R., Zeng Y., Liang J., Zhang H., Yi J., Liu Z. (2024). Effect of *Rhodotorula mucilaginosa* inoculation on the aroma development of a fermented vegetables simulated system. Food Research International.

[bb0175] Xue B.J., You Y.M., Du M.Y., Ibrahim A., Suo H.Y., Zhang F.S., Zheng J. (2025). Metagenomic analysis of *Lactobacillus plantarum* DACN768 inoculation effects on volatile flavor compounds, microbial succession, and flavor metabolic network in suansun. Food Research International.

[bb0180] Xue Y.Q., Chen J., Wang L., Wang Y.W., Xu F. (2024). Exploring the flavor changes in mung bean flour through *Lactobacillus* fermentation: Insights from volatile compounds and non-targeted metabolomics analysis. Journal of the Science of Food and Agriculture.

[bb0185] Yang M., Huang J., Zhou R., Qi Q., Peng C., Zhang L., Wu C. (2021). Characterizing the microbial community of Pixian doubanjiang and analysing the metabolic pathway of major flavour metabolites. LWT- Food Science and Technology.

[bb0190] Yang S.J., Bai M., Kwok L.Y., Zhong Z., Sun Z.H. (2025). The intricate symbiotic relationship between lactic acid bacterial starters in the milk fermentation ecosystem. Critical Reviews in Food Science and Nutrition.

[bb0195] Yao W.T., Yang L.Z., Shao Z.H., Xie L., Chen L.M. (2020). Identification of salt tolerance-related genes of *Lactobacillus plantarum* D31 and T9 strains by genomic analysis. Annals of Microbiology.

[bb0200] Yi J., Kebede B., Kristiani K., Buve C., Van Loey A., Grauwet T., Hendrickx M. (2018). The potential of kiwifruit puree as a clean label ingredient to stabilize high pressure pasteurized cloudy apple juice during storage. Food Chemistry.

[bb0205] Yin X., Li J., Shin H.-D., Du G., Liu L., Chen J. (2015). Metabolic engineering in the biotechnological production of organic acids in the tricarboxylic acid cycle of microorganisms: Advances and prospects. Biotechnology Advances.

[bb0210] Zhang L., Che Z., Xu W., Yue P., Li R., Li Y., Zeng P. (2020). Dynamics of physicochemical factors and microbial communities during ripening fermentation of Pixian doubanjiang, a typical condiment in Chinese cuisine. Food Microbiology.

[bb0215] Zhang L., Ma H., Kulyar M.F.-E.A., Pan H., Li K., Li A., Li J. (2022). Complete genome analysis of *Lactobacillus fermentum* YLF016 and its probiotic characteristics. Microbial Pathogenesis.

[bb0220] Zhang S., Shang Z., Liu Z., Hu X., Yi J. (2023). Flavor production in fermented chayote inoculated with lactic acid bacteria strains: Genomics and metabolomics based analysis. Food Research International.

[bb0225] Zhao C.J., Schieber A., Gaenzle M.G. (2016). Formation of taste-active amino acids, amino acid derivatives and peptides in food fermentations - a review. Food Research International.

[bb0230] Zhao H.Y., Liu L.X., Peng S., Yuan L., Li H., Wang H. (2019). Heterologous expression of argininosuccinate synthase from oenococcus oeni enhances the acid resistance of *Lactobacillus plantarum*. Frontiers in Microbiology.

[bb0235] Zhao J.X., Dai X.J., Liu X.M., Zhang H., Tang J.A., Chen W. (2011). Comparison of aroma compounds in naturally fermented and inoculated Chinese soybean pastes by GC-MS and GC-olfactometry analysis. Food Control.

[bb0240] Zhao S., Hao C., Niu C., Lu J., Wang L., Shi Y., Li Q. (2024). Insight into the keystones of Chinese broad bean paste fermentation: Brewing techniques, chemosensory characteristics, and microbial community. Trends in Food Science & Technology.

[bb0245] Zhou Z., Wang X., Duan C., Liu Z., Wang Y., Zhong Y., Wang T. (2024). A synergistic fermentation system of probiotics with low-cost and high butyric acid production: *Lactiplantibacillus plantarum* and *clostridium tyrobutyricum*. Food Bioscience.

