# Supplementary material for: Effects of a novel salt-tolerance Lactiplantibacillus plantarum on the physicochemical properties and volatile profiles of broad-bean paste
Source: Food Chem X. 2026 Jan 26;34:103598. doi: 10.1016/j.fochx.2026.103598 (PMC12874603; doi:10.1016/j.fochx.2026.103598)
Supplement: Supplementary file 1 — Supplementary material [file mmc1.docx]

Table S1. Free amino acids content in fermented broad bean paste juice.

| **Flavor** | **Amino acid** | **Concentration (g/L)** | | | |
| --- | --- | --- | --- | --- | --- |
|  |  | **Unsterilized** | | **Sterilization** | |
|  |  | **NF** | **LPI** | **SC** | **SLPI** |
| **Sweet** | L-Alanine | 5.07 ± 0.49^c^ | 2.82 ± 0.09^d^ | 9.95 ± 0.51^a^ | 6.05 ± 0.11^b^ |
|  | L-Proline | 4.84 ± 0.46^c^ | 2.74 ± 0.06^d^ | 9.46 ± 0.50^a^ | 5.95 ± 0.20^b^ |
|  | Serine | 1.73 ± 0.04^b^ | 1.55 ± 0.00^c^ | 1.89 ± 0.01^a^ | 1.74 ± 0.02^b^ |
|  | L-Threonine | 1.32 ± 0.19^ab^ | 1.49 ± 0.09^a^ | 0.78 ± 0.06^c^ | 1.04 ± 0.18^bc^ |
|  | L-Methionine | 0.26 ± 0.01^b^ | 0.25 ± 0.02^c^ | 0.31 ± 0.00^a^ | 0.11 ± 0.01^d^ |
|  | Glycine | 0.11 ± 0.00^a^ | 0.11 ± 0.04^a^ | 0.14 ± 0.00^a^ | 0.11 ± 0.00^a^ |
|  | Beta-Alanine | 0.01 ± 0.00^a^ | 0.01 ± 0.01^a^ | 0.02 ± 0.00^a^ | 0.01 ± 0.00^a^ |
| **Umami** | L-Glutamic acid | 2.43 ± 0.32^a^ | 2.29 ± 0.36^ab^ | 1.48 ± 0.06^c^ | 1.94 ± 0.05^bc^ |
|  | L-Aspartic acid | 0.89 ± 0.01^c^ | 0.85 ± 0.02^c^ | 1.14 ± 0.05^a^ | 0.97 ± 0.04^b^ |
| **Bitter** | L-Lysine | 4.32 ± 0.11^a^ | 3.95 ± 0.14^b^ | 3.95 ± 0.05^b^ | 3.67 ± 0.09^c^ |
|  | L-Arginine | 2.78 ± 0.10^ab^ | 2.89 ± 0.23^a^ | 2.65 ± 0.03^ab^ | 2.51 ± 0.09^b^ |
|  | L-Valine | 2.49 ± 0.11^a^ | 2.36 ± 0.18^a^ | 2.33 ± 0.06^a^ | 2.32 ± 0.15^a^ |
|  | L-Phenylalanine | 1.51 ± 0.03^a^ | 1.43 ± 0.10^a^ | 1.45 ± 0.03^a^ | 1.40 ± 0.05^a^ |
|  | L-Histidine | 0.61 ± 0.02^b^ | 0.56 ± 0.03^b^ | 0.64 ± 0.01^a^ | 0.54 ± 0.00^b^ |
|  | L-Tyrosine | 0.20 ± 0.00^b^ | 0.19 ± 0.02^b^ | 0.36 ± 0.01^a^ | 0.35 ± 0.03^b^ |
|  | L-Tryptophan | 0.0015 ± 0.0000^b^ | 0.0016 ± 0.0002^b^ | 0.0020 ± 0.0000^a^ | 0.0005 ± 0.0001^c^ |
| **Others** | L-Citrulline | 4.52 ± 0.38^a^ | 4.68 ± 0.58^a^ | 4.38 ± 0.12^a^ | 2.88 ± 0.12^b^ |
|  | L-Asparagine | 3.31 ± 0.28^a^ | 3.54 ± 0.38^a^ | 3.72 ± 0.15^a^ | 3.16 ± 0.17^a^ |
|  | L-Glutamine | 0.36 ± 0.02^a^ | 0.36 ± 0.06^a^ | 0.02 ± 0.00^b^ | 0.02 ± 0.00^b^ |
|  | L-Ornithine | 0.28 ± 0.00^c^ | 0.26 ± 0.04^c^ | 0.40 ± 0.01^a^ | 0.36 ± 0.00^b^ |
|  | 4-Aminobutyric acid | 0.08 ± 0.01^a^ | 0.08 ± 0.00^a^ | 0.07 ± 0.00^a^ | 0.07 ± 0.00^a^ |
|  | 4-Hydroxyproline | 0.0113 ± 0.0011^a^ | 0.0150 ± 0.0047^a^ | 0.0122 ± 0.0004^a^ | 0.0103 ± 0.0018^a^ |
|  | 1-Methyhistidine | 0.0012 ± 0.0000^a^ | 0.0011 ± 0.0002^a^ | 0.0011 ± 0.0000^ab^ | 0.0009 ± 0.0000^b^ |
|  | 5-Hydroxylysine | 0.0007 ± 0.0000^b^ | 0.0007 ± 0.0001^b^ | 0.0004 ± 0.0000^c^ | 0.0009 ± 0.0000^a^ |
|  | 3-Methyhistidine | 0.0006 ± 0.0000^a^ | 0.0006 ± 0.0001^a^ | 0.0006 ± 0.0000^a^ | 0.0005 ± 0.0000^a^ |

Note: Different superscript letters (a, b, c) within the same row denote statistically significant differences (*p* < 0.05) between treatment groups after Holm-Šidák correction for multiple comparisons; NF, natural fermentation; LPI, inoculated with *L. plantarum* YQ1; SC, sterilized control; SLPI, sterilized and inoculated with *L. plantarum* YQ1.

Table S2. Concentrations of key volatile flavor compounds in fermented broad bean paste juice.

| **Chemical Class** | **Volatile Compound** | **RI** | **RI_0_** | **Concentration(μg/L)** | | | | **Contribution to flavor** |
| --- | --- | --- | --- | --- | --- | --- | --- | --- |
|  |  |  |  | **NF** | **LPI** | **SC** | **SLPI** |  |
| **Alcohols** | 3-Methyl-1-butanol | 720 | 726 | 27.76 ± 8.35^b^ | 89.93 ± 39.90^a^ | ND | ND | Fruity and banana-like sweet |
|  | 2,3-Butanediol | 782 | 775 | 157.74 ± 10.05^a^ | 49.28 ± 0.37^b^ | 52.80 ± 16.44^b^ | 55.98 ± 9.51^b^ | Mild, sweet, buttery |
|  | 2-Furanmethanol | 848 | 850 | 384.27 ± 10.77^b^ | 221.16 ± 17.66^c^ | 761.41 ± 86.60^a^ | 332.60 ± 29.41^b^ | Caramel, sweet, bready |
|  | 1-Octen-3-ol | 981 | 981 | 28.20 ± 2.77^a^ | 28.33 ± 2.70^a^ | ND | ND | Mushroom-like |
|  | Benzyl alcohol | 1033 | 1033 | 11.95 ± 4.80^b^ | 78.10 ± 13.11^a^ | 19.61 ± 2.49^b^ | 15.12 ± 1.74^b^ | Floral, mild, slightly sweet |
|  | (E)-2-Octen-1-ol | 1068 | 1069 | 12.42 ± 1.09^a^ | 10.50 ± 1.07^b^ | ND | ND | Fatty, green, slightly metallic |
|  | Linalool | 1100 | 1101 | ND | 1.30 ± 0.17^b^ | 10.59 ± 0.70^a^ | 1.83 ± 1.90^b^ | Citrus-like, floral |
|  | Phenylethyl alcohol | 1110 | 1110 | 108.10 ± 27.75^b^ | 579.82 ± 80.40^a^ | 35.66 ± 4.08^bc^ | 19.46 ± 3.04^c^ | Floral, rose-like, sweet |
|  | α-Terpineol | 1193 | 1190 | 1.60 ± 0.24^b^ | 1.38 ± 0.08^b^ | 9.84 ± 2.20^a^ | 3.31 ± 0.79^b^ | Pine, lilac, mild citrus |
| **Esters** | 3-Hydroxy-butanoic acid ethyl ester | 934 | 915 | 0.33 ± 0.19^a^ | 0.23 ± 0.06^b^ | ND | ND | Sweet fruity, creamy, caramel-like |
|  | Dimethyl phthalate | 1444 | 1454 | 1.76 ± 0.47^ab^ | 1.92 ± 0.20^a^ | 1.26 ± 0.31^bc^ | 0.93 ± 0.05^c^ | Nearly odorless |
|  | Tetradecanoic acid ethyl ester | 1792 | 1794 | ND | 0.21 ± 0.00^a^ | ND | ND | Fruity, floral, slightly fatty |
|  | Hexadecanoic acid ethyl ester | 1991 | 1991 | 0.68 ± 0.47^a^ | 0.64 ± 0.26^a^ | 0.23 ± 0.06^b^ | ND | Creamy, faint fruity |
| **Aldehydes** | Furfural | 830 | 823 | 73.34 ± 0.98^b^ | ND | 200.50 ± 8.07^a^ | 60.66 ± 12.40^b^ | Sweet caramel, toasty, nutty |
|  | Benzaldehyde | 959 | 959 | 309.98 ± 25.75^a^ | 40.35 ± 20.02^c^ | 195.65 ± 21.18^b^ | 277.74 ± 7.30^a^ | Bitter almond-like, marzipan-like |
|  | Phenylacetaldehyde | 1043 | 1043 | 185.28 ± 1.15^b^ | 110.87 ± 15.32^c^ | 239.59 ± 33.70^a^ | 143.63 ± 9.40^c^ | Floral honey-like |
| **Ketones** | 1-(2-Furanyl)-ethanone | 905 | 900 | ND | ND | 141.63 ± 24.52^a^ | 54.77 ± 16.23^b^ | Caramel-coffee, roasted nuts, soy sauce-like |
|  | 1-(2-Furanyl)-1-propanone | 1004 | 1016 | ND | ND | 25.13 ± 3.37^a^ | 12.73 ± 4.13^b^ | Caramel-sweet, maple syrup, nutty |
|  | Acetophenone | 1063 | 1063 | 7.32 ± 0.82^a^ | 3.89 ± 2.17^b^ | ND | ND | Bitter almond, cherry-like, floral |
|  | β-Damascenone | 1376 | 1384 | ND | ND | 6.21 ± 2.36^a^ | 2.40 ± 0.28^b^ | Cooked apple-like |
|  | Geranylacetone | 1446 | 1444 | ND | ND | 1.53 ± 0.39^a^ | 0.90 ± 0.14^b^ | Fruit |
| **Acids** | 3-Methyl-butanoic acid | 863 | 873 | 804.69 ± 6.62^a^ | 151.45 ± 70.54^c^ | 392.40 ± 95.19^b^ | 150.97 ± 22.92^c^ | Pungent cheesy, parmesan-like |
|  | 2-Methyl-butanoic acid | 901 | 876 | 40.14 ± 2.05^a^ | 15.09 ± 15.45^b^ | 23.75 ± 6.16^a^ | 14.78 ± 0.98^b^ | Fermented cheese, fruity |
|  | 3-Methyl-pentanoic acid | 951 | 951 | 4.71 ± 0.69^a^ | ND | ND | ND | Rancid fat, persistent sweaty odor |
| **Pyrazines** | Methylpyrazine | 816 | 820 | ND | ND | 21.89 ± 8.53^a^ | 23.78 ± 8.35^a^ | Roasted nuts, coffee, cocoa |
|  | 2,5-Dimethylpyrazine | 911 | 911 | ND | ND | 97.11 ± 16.84^a^ | 103.87 ± 17.10^a^ | Nutty, roasted, popcorn-like |
|  | 2,6-Dimethylpyrazine | 912 | 919 | 9.55 ± 0.90^a^ | 3.97 ± 0.06^b^ | ND | 0.65 ± 0.42^c^ | Nutty roast, cocoa, earthy |
|  | 2-Ethyl-5-methylpyrazine | 996 | 995 | ND | ND | 17.54 ± 2.60^a^ | ND | Offee, toasted hazelnut, caramel |
|  | Acetylpyrazine | 1019 | 1029 | ND | ND | 8.42 ± 1.30^a^ | ND | Popcorn, roasted peanuts, crusty bread |
|  | 3-Ethyl-2,5-dimethylpyrazine | 1076 | 1074 | ND | ND | ND | 5.21 ± 1.42^a^ | Meaty roast, potato chips, smoky |
| **Others** | Dihydro-2-methyl-3(2H)-furanone | 802 | 808 | ND | ND | 17.58 ± 8.38^a^ | 4.91 ± 2.02^b^ | Caramel-sweet, maple syrup, nutty |
|  | 3-(Methylthio) propionaldehyde | 905 | 905 | 71.47 ± 9.53^a^ | ND | ND | ND | Boiled potato, tomato soup |
|  | 5-Ethyldihydro-2(3H)-furanone | 1049 | 1038 | 1.68 ± 0.15^a^ | 1.14 ± 0.64^a^ | ND | 0.22 ± 0.14^b^ | Creamy coconut, peach-apricot, waxy |
|  | 1-(1H-Pyrrol-2-yl)-ethanone | 1071 | 1059 | ND | ND | 727.09 ± 29.02^a^ | 435.62 ± 60.66^b^ | Popcorn roast, nutty (almond), earthy-musty |
|  | cis-5-Ethenyltetrahydro-α,α,5-trimethyl-2-furanmethanol | 1086 | 1086 | 1.23 ± 0.19^a^ | 1.66 ± 0.45^a^ | ND | ND | Caramel-coffee, brown sugar, woody-smoky |
|  | Benzyl nitrile | 1135 | 1150 | 9.86 ± 0.46^b^ | 7.53 ± 0.95^bc^ | 13.71 ± 2.33^a^ | 5.74 ± 1.37^c^ | Almond-like, bitter, sharp |
|  | 4-Ethyl-2-methoxy-Phenol | 1270 | 1268 | ND | ND | ND | 1.37 ± 0.35^a^ | Smoky bacon, charred wood, clove spice |
|  | Anethole | 1283 | 1281 | ND | ND | 43.25 ± 1.56^a^ | 23.94 ± 3.80^b^ | Licorice-sweet, star anise, floral hint |
|  | Eugenol | 1348 | 1348 | 50.45 ± 0.09^a^ | 50.36 ± 4.41^a^ | 41.53 ± 7.33^a^ | 25.01 ± 3.02^b^ | Clove spice, cinnamon warmth |
|  | γ-Decalactone | 1356 | 1356 | 9.26 ± 0.63^a^ | 9.22 ± 0.79^a^ | 9.18 ± 0.84^a^ | 6.05 ± 1.08^b^ | Intense peach, creamy coconut, buttery-sweet |
|  | Methyleugenol | 1412 | 1405 | ND | ND | 0.89 ± 0.29^a^ | ND | Ripe banana, clove-like, woody |
|  | Caryophyllene oxide | 1555 | 1558 | ND | 0.19 ± 0.04^a^ | 0.06 ± 0.01^b^ | ND | Woody spice |

Note: Different superscript letters (a, b, c) within the same row denote statistically significant differences (p < 0.05) between treatment groups after Holm-Šidák correction for multiple comparisons; NF, natural fermentation; LPI, inoculated with *L. plantarum* YQ1; SC, sterilized control; SLPI, sterilized and inoculated with *L. plantarum* YQ1; ND denotes that the substance was not detected; RI denotes the calculated retention index; RI0 denotes the reference retention index. Reference indices were obtained from the NIST Chemistry WebBook (SRD 69) [URL: <http://webbook.nist.gov/chemistry/>].
